# Effect of prenatal glucocorticoids and thyroid hormones on developmental plasticity of mitochondrial aerobic metabolism, growth and survival: an experimental test in wild great tits

**DOI:** 10.1242/jeb.243414

**Published:** 2022-05-10

**Authors:** Nina Cossin-Sevrin, Bin-Yan Hsu, Coline Marciau, Vincent A. Viblanc, Suvi Ruuskanen, Antoine Stier

**Affiliations:** ^1^ Department of Biology, University of Turku, FI-20014 Turku, Finland; ^2^ Université de Strasbourg, Centre National de la Recherche Scientifique, Institut Pluridisciplinaire Hubert Curien, UMR 7178, 67087 Strasbourg, France; ^3^ Institute for Marine and Antarctic Studies, University of Tasmania, Battery Point, TAS 7004, Australia; ^4^ Department of Biological and Environmental Sciences, University of Jyväskylä, FI-40014 Jyväskylä, Finland; ^5^ Université Claude Bernard Lyon 1, CNRS, ENTPE, UMR 5023 LEHNA, F-69622 Villeurbanne, France

**Keywords:** Cellular metabolism, Corticosterone, Prenatal programming, Avian development, Thyroid hormones, *Parus major*

## Abstract

Developmental plasticity is partly mediated by transgenerational effects, including those mediated by the maternal endocrine system. Glucocorticoid and thyroid hormones may play central roles in developmental programming through their action on metabolism and growth. However, the mechanisms by which they affect growth and development remain understudied. One hypothesis is that maternal hormones directly affect the production and availability of energy-carrying molecules (e.g. ATP) by their action on mitochondrial function. To test this hypothesis, we experimentally increased glucocorticoid and thyroid hormones in wild great tit eggs (*Parus major*) to investigate their impact on offspring mitochondrial aerobic metabolism (measured in blood cells), and subsequent growth and survival. We show that prenatal glucocorticoid supplementation affected offspring cellular aerobic metabolism by decreasing mitochondrial density, maximal mitochondrial respiration and oxidative phosphorylation, while increasing the proportion of the maximum capacity being used under endogenous conditions. Prenatal glucocorticoid supplementation only had mild effects on offspring body mass, size and condition during the rearing period, but led to a sex-specific (females only) decrease in body mass a few months after fledging. Contrary to our expectations, thyroid hormone supplementation did not affect offspring growth or mitochondrial metabolism. Recapture probability as juveniles or adults was not significantly affected by prenatal hormonal treatment. Our results demonstrate that prenatal glucocorticoids can affect post-natal mitochondrial density and aerobic metabolism. The weak effects on growth and apparent survival suggest that nestlings were mostly able to compensate for the transient decrease in mitochondrial aerobic metabolism induced by prenatal glucocorticoids.

## Teaser

**Summary:** Mitochondrial aerobic metabolism is influenced by prenatal glucocorticoid exposure in a wild passerine bird species, with no evident consequences for growth rate or survival.

## INTRODUCTION

Genetic inheritance has long dominated evolutionary thinking (Pigliucci, 2007). Yet, recent advances in evolutionary biology are calling for an extension of this framework and are emphasizing the role of complementary mechanisms (e.g. epigenetic status; transmission of substances such as hormones or RNA; transmission of nutrients) (Bonduriansky and Day, 2009; Forsman, 2015; Laland et al., 2015; Müller, 2017; Pigliucci, 2007). Developmental plasticity, in particular, occurs when environmental conditions during ontogenesis create anatomical, physiological and behavioral changes in individual phenotypes that remain throughout life (Piersma and Gils, 2011). This plasticity can be a direct response to prevailing environmental conditions, but also the consequence of parental effects, which can themselves be a response to current environmental conditions (Proulx and Teotónio, 2017; Uller, 2008). In this case, offspring phenotype is not only determined by its own environment and genotype, and the interactions between the two, but also by the environment and characteristics of its parents, a phenomenon referred to as intergenerational or transgenerational plasticity (Marshall and Uller, 2007). Maternal effects, in particular, represent a major pathway in transgenerational developmental plasticity. They rely on diverse mechanisms, such as nutrient transfer or maternally inherited epigenetic modifications (Alfaradhi and Ozanne, 2011; Laland et al., 2015; Myatt, 2006).

The endocrine system, in particular, is a key mediator of maternal effects on developmental plasticity (Dufty et al., 2002; Fowden and Forhead, 2009; Groothuis et al., 2005). Hormone transfer from mother to offspring can have important effects on offspring traits, including effects on the development and growth of juveniles (Groothuis et al., 2019; Meylan et al., 2012). This is particularly true during the initial stages of development when offspring rely on maternally transferred hormones, before starting their own endogenous hormone production with a fully developed endocrine system (Darras, 2019; McNabb, 2006; Schwabl, 1999). Variation in hormone levels promotes developmental plasticity through changes in gene expression, modifying a wide array of physiological, behavioral and morphological traits (e.g. begging behavior, immune function; Groothuis et al., 2005), including metabolic rate (e.g. through transcription factors, cell signaling, growth factors; Dufty et al., 2002; Meylan et al., 2012).

Whereas the effects of maternal androgens (e.g. testosterone, 5α-dihydrotestosterone, andostenedione) on offspring development have been well studied (Groothuis et al., 2005; Podmokła et al., 2018), less is known about the effects of thyroid hormones (THs). Yet, THs are central growth regulators, and coordinate maturation and differentiation as transcription factors (Darras, 2019; Ruuskanen and Hsu, 2018). Thus, variation in THs during critical periods may have marked effects on offspring development (e.g. neurotrophic signals, cerebellum-mediated motor function, retinal layer) (Darras, 2019; Ruuskanen and Hsu, 2018), and is also known to affect offspring behavior via early-life imprinting (Bett et al., 2016; Yamaguchi et al., 2012). THs modulate metabolism associated with (i) medium to long-term changes in the basal energy expenditure of the organism (Harper and Seifert, 2008; Kim, 2008) and (ii) modulation of the activity of downstream regulatory hormones and growth factors such as insulin, glucagon and catecholamines (Grøntved et al., 2015; Pucci et al., 2000; Sinha et al., 2018).

Glucocorticoid hormones (glucocorticoids, GCs) are another well-known regulator of metabolic (Rose et al., 2010) and developmental processes (Miyazawa and Aulehla, 2018; Rieger, 1992). Prenatal GCs play a role in offspring developmental plasticity (Seckl, 2004), and GC-mediated maternal effects potentially lead to long-lasting changes in offspring phenotype and metabolism (e.g. neurodevelopmental and cardio-metabolic effects; Aghajafari et al., 2002; Eberle et al., 2021). GCs have been shown to modulate the expression of up to 10% of the genome (Le et al., 2005; Xavier et al., 2016). As direct regulators of metabolic processes, GCs also enable the organism to accommodate changes in energetic demands through a variety of mechanisms (ranging from appetite to glycogenolysis and lipolysis regulation; Rose et al., 2010; Sapolsky et al., 2000). The impact of GCs on metabolism is often investigated from the point of view of individual responses to stress (i.e. as the consequence of stress-induced changes in GC levels; Crespi et al., 2013), though GCs primarily play a role in regulating body homeostasis (MacDougall-Shackleton et al., 2019).

At the same time, a growing body of evidence is pointing towards mitochondrial function (the primary role of which is to transduce energy acquired from nutrients into ATP) as the central link between the endocrine system, metabolism and growth (Koch et al., 2021; Picard et al., 2014; Salin et al., 2019). Specifically, THs have been shown to modulate mitochondrial activity both directly (Cioffi et al., 2013; Noli et al., 2020) and indirectly by up-regulating mitochondrial biogenesis (Weitzel and Iwen, 2011). Short- and long-term exposure to low physiological amounts of GCs also enhances mitochondrial function (as measured through membrane potential, proton leak, ATP production or maximal mitochondrial capacity), while chronic exposure to high levels of corticosterone may decrease it (Casagrande et al., 2020; Manoli et al., 2007; Picard et al., 2014). Thus, we may expect the impact of maternal effects on offspring phenotype (e.g. growth) to be mediated by the action of prenatal maternal hormones on mitochondrial function. There is growing evidence that despite flexibility in mitochondrial function, stable inter-individual differences through time exist (e.g. Braganza et al., 2020; Stier et al., 2019; Stier et al., 2022). Inter-individual differences might arise from developmental plasticity (Gyllenhammer et al., 2020; Stier et al., 2022). Yet, to the best of our knowledge, very little is known about the impact of prenatal hormones in shaping offspring mitochondrial function (but see Davies et al., 2021; Grilo et al., 2021).

**Fig. 1.**
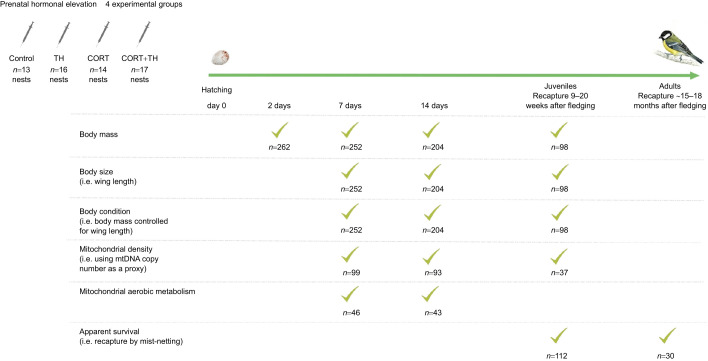
**Experimental time line of the study.** Sample sizes are shown for the different response variables. Great tit nestlings fledge around 18–20 days after hatching. TH, thyroid hormone; CORT, corticosterone.

The purpose of our study was to investigate the effects of prenatal exposure to elevated levels of THs and GCs on offspring mitochondrial aerobic metabolism, growth and survival throughout postnatal development. We aimed at mimicking an increase in maternal TH and GC levels deposited in the eggs by experimentally injecting eggs of wild great tit (*Parus major*) before the onset of incubation with physiological doses of THs and/or GCs, or with saline solution (control), in a controlled full-factorial (2×2) study design. We assessed differences between individuals hatching from treated and control eggs in terms of embryonic development duration, body size, body mass, body condition (body mass adjusted for size) and changes in blood cell mitochondrial density and respiration. We evaluated effects on offspring from hatching (day 2) through to fledging (day 14), with an intermediate measure performed at day 7 (see Fig. 1 for the experimental time line and sample size). We also recaptured a fraction of the birds as juveniles (ca*.* 9–20 weeks after fledging) and as adults (ca*.* 15–18 months after fledging) and tested for the consequences of elevated prenatal hormone levels on short-term (fledging), medium-term (first autumn after fledging) and long-term (second autumn after fledging) survival (using recapture probability as a proxy).

**Fig. 2.**
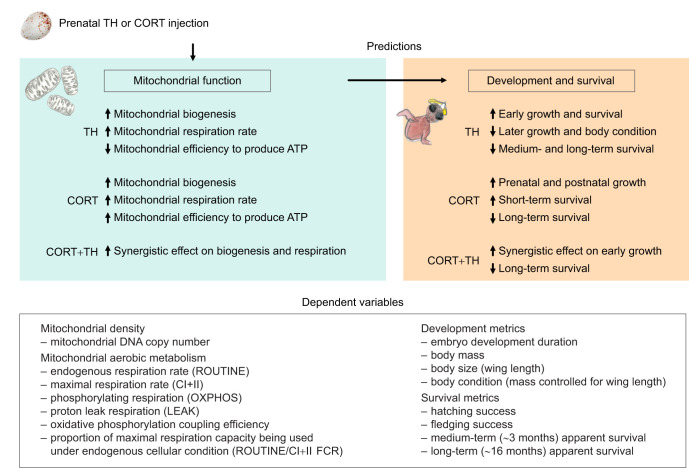
**Predictions related to the experimental manipulation of prenatal TH and CORT.** Hormone injection increased the yolk content of TH and CORT by 2 s.d.

As THs are known to stimulate mitochondrial aerobic metabolism and biogenesis while potentially decreasing the efficiency at which nutrients are converted to ATP (Cioffi et al., 2013), we expected nestlings hatched from eggs supplemented with THs to exhibit a higher mitochondrial density and higher mitochondrial respiration rates, but a potentially higher proton leak, leading to less efficient mitochondria (Fig. 2). We predicted that such a higher metabolic capacity could boost embryo development and early post-hatching growth and survival, while the lower mitochondrial efficiency might impair body condition and performance later during postnatal development (Salin et al., 2019), leading to a decrease in survival prospects especially after fledging (but see Hsu et al., 2019, 2020, 2021 preprint; Ruuskanen et al., 2016; Sarraude et al., 2020, for the contrasting effects of prenatal THs on growth in avian species). As physiological amounts of GC have been suggested to enhance mitochondrial density and aerobic metabolism (including ATP production; Manoli et al., 2007), we expected nestlings hatched from eggs supplemented with GCs to exhibit a higher mitochondrial density and higher mitochondrial respiration rate, as well as a higher efficiency of ATP production (Fig. 2; but see Casagrande et al., 2020, for somewhat opposite effects of high GC levels at the postnatal stage). Thus, we expected these individuals to have a faster growth (both prenatal and postnatal), leading to an increase in survival prospects in the short term (i.e. fledging and/or first autumn) but with potential long-term costs (Haussmann et al., 2012; Metcalfe and Monaghan, 2001). Finally, we tested for interactions between GCs and THs, such as synergistic effects, affecting offspring mitochondrial function, growth and survival (Brown et al., 2014). For instance, it has been shown that postnatal supplementation with THs and GCs has synergistic effects on growth (Khangembam et al., 2017). However, directional predictions about the effects of prenatal hormones are very difficult to make considering (1) the likely environmental dependence of their cost–benefit balance, (2) the existence of non-linear dose–responses and (3) the fact that embryos are not passive receivers of maternal hormones but can manipulate such signals (Groothuis et al., 2019).

## MATERIALS AND METHODS

### Field site and population monitoring

The study was conducted on a population of wild great tits (*Parus major* Linnaeus 1758) breeding in artificial nest boxes (*n*=374) on Ruissalo Island, Finland (60°26.055′N, 22°10.391′E). The data were collected during the 2019 breeding season (April to July), and during the autumn of 2019 and of 2020 (October to November). Nest boxes were checked every 5 days during the breeding season to monitor occupation. We also recorded the date of laying the first egg (laying date), incubation onset, clutch size, hatching date (±24 h), developmental duration (±24 h; i.e. the time between incubation onset and hatching), brood size and fledging success.

### Experimental manipulation of GCs and THs

To manipulate the prenatal hormonal environment that offspring were exposed to, nests were randomly divided into 4 groups, and eggs received (i) an injection of control isotonic saline solution (control, 2 µl NaCl), (ii) an injection elevating TH (a mixture of 0.325 ng T4 and 0.041 ng T3 per yolk), (iii) an injection elevating corticosterone (CORT; 0.202 ng per yolk) or (iv) an injection elevating both CORT and TH (i.e. 0.325 ng of T4+0.041 ng of T3+0.202 ng of CORT). Our objective was to increase yolk hormone content by 2 s.d. while remaining in their natural physiological range, as recommended by Podmokła et al. (2018). Based on the literature and hormonal measurements from the same population, average TH content in great tits is expected to be (mean±s.d.) 0.053±0.020 ng per yolk for T3 and 0.458±0.162 ng per yolk for T4 (Ruuskanen et al., 2018), while average CORT content is expected to be 0.215±0.101 ng per yolk (based on the averages for great tits from Groothuis and Schwabl, 2008; Lessells et al., 2016; Mentesana et al., 2019; Groothuis and Schwabl, 2008; Mentesana et al., 2019; Lessells et al., 2016, calculated using an average yolk mass of 315 mg as in Lessells et al., 2016).

Hormone solutions were prepared using T4 (l-thyroxine 98% HPCL, CAS number 51-48-9, Sigma-Aldrich), T3 (3,3′,5-triiodo-l-thyronine, >95% HPCL, CAS number 6893-02-3, Sigma-Aldrich) and CORT (Corticosterone VETRANAL^®^, HPCL, CAS number 50-22-6, Sigma-Aldrich) dissolved in 0.1 mol l^−1^ NaOH (TH) or 99% ethanol (CORT), and diluted in 0.9% NaCl to the targeted concentrations. We followed the injection procedure as described in Hsu et al. (2019) and Sarraude et al. (2020). We prepared the corresponding hormone solutions for each experimental group (control, TH, CORT or CORT+TH), so that each egg was injected only once with 2 µl of the corresponding hormone solution and all eggs in one nest received the same hormonal mix. Egg injections started on the day the 5th egg was laid, and continued every day after until the last egg was laid. This protocol ensured injections were done before incubation onset, meanwhile minimizing nest disturbance (i.e. we avoided visiting the nest every day) and allowing us to closely monitor the onset of incubation, given that great tits can start incubation well before clutch completion. When no new eggs were observed for two consecutive days, the clutch was considered complete. Hatching was monitored daily starting 2 days prior to the estimated hatch date. Hatching was considered as ‘day 0’.

Nestlings were individually marked (nail-clipping at day 2, metal ring at day 7), weighed with an electronic scale (body mass ±0.1 g) at 2, 7 and 14 days, and measured with a metal ruler (wing length ±1 mm) at 7 and 14 days (see Fig. 2 for a time line of the study). Nestlings fledge around 18–20 days. When recaptured in the following autumns (see below), body mass and wing length were measured. We also blood sampled individuals (∼30–75 µl from the brachial vein using heparinized capillaries) at 7 and 14 days and as juveniles the following autumn. Blood samples were used to measure mitochondrial DNA (mtDNA) copy number (an index of mitochondrial density, see below) and evaluate mitochondrial aerobic metabolism in 7 and 14 day old nestlings (Fig. 2). The use of blood samples has the advantage of being minimally invasive, allowing the longitudinal sampling of individuals (Koch et al., 2021; Stier et al., 2017).

We recaptured nestlings from the experiment as juveniles the following autumn (in 2019, i.e. between 9 and 20 weeks after fledging). For this, we used mist-nests with playback at seven feeding stations in the study plots (3 h per feeding station on 3 separate days over 2 months, summing up to a total of 100 h of mist-netting). If a bird was recaptured several times during this period, only the measurements from the first capture were used for body mass, body size and blood samples. Nestlings were also recaptured as adults (i.e. between 15 and 18 months after fledging) using a similar method (six feeding stations, a total of 95 h of mist-netting) in autumn 2020. In addition, we included recapture data from a mist-netting site (Ruissalo botanical garden; 3 km from the study plots) where mist-netting was conducted regularly throughout the year every 1 or 2 weeks (4 h per session). Data collected from the 2019 recapture sessions were used to analyze juvenile body mass, size and condition and mitochondrial DNA copy number, and for estimating recapture probability a few months after fledging (i.e. used here as a proxy of medium-term apparent survival). Data collected from autumn 2020 trapping sessions and continuous mist-netting were used as a proxy of long-term survival (i.e. recapture probability during and after the first winter experienced by juveniles).

In total, the experiment included 60 great tit nests, resulting in 468 injected eggs (eggs/nests: *n*_control_=108/13_,_ *n*_TH_=118/16, *n*_CORT_=111/14, *n*_CORT+TH_=131/17) and 267 chicks being monitored (nestlings/nests: *n*_control_=60/12_,_ *n*_TH_=75/15, *n*_CORT_=58/13, *n*_CORT+TH_=74/13); 112 juveniles were caught in the autumn of 2019 (juveniles/nests: *n*_control_=25/10_,_ *n*_TH_=22/9, *n*_CORT_=28/10, *n*_CORT+TH_=37/10) and 30 adults in the autumn of 2020 (adults/nests: *n*_control_=6/5_,_ *n*_TH_=6/5, *n*_CORT_=6/5, *n*_CORT+TH_=12/8).

### mtDNA copy number

We randomly selected 2 nestlings per nest (*n*=104 individuals) and estimated mtDNA copy number on the same individuals at day 7, day 14 and as juveniles (autumn 2019) when samples were available (sample sizes at day 7, day 14 and juveniles, respectively: *n*_control_=26/27/9, *n*_CORT_=23/21/10, *n*_TH_=29/24/7, *n*_CORT+TH_=25/23/11, resulting in 235 samples in total). Genomic DNA was extracted from 5 µl of frozen blood samples using a salt extraction procedure adapted from Aljanabi and Martinez (1997). DNA quantity and purity were estimated using a NanoDrop spectrophotometer*.* Samples were re-extracted if needed ([DNA]<50 ng µl^−1^, *A*_260/280_<1.80 or *A*_260/230_<2). DNA integrity of 48 randomly selected samples was evaluated and deemed satisfactory using gel electrophoresis (100 ng of DNA, Midori Green staining, 0.8% agarose gel at 100 mV for 60 min). Samples meeting our quality checks were then diluted at 1.2 ng μl^−1^ in sterile H_2_O and stored at −80°C until real-time quantitative PCR (qPCR) analysis. mtDNA copy number was quantified using qPCR as previously described for other passerine species (Stier et al., 2019, 2020) including great tits (Hsu et al., 2021 preprint; Stier et al., 2021 preprint). This technique estimates the relative mtDNA copy number by determining the ratio of mtDNA repeat copy number to a nuclear singly copy gene (SCG). qPCR reactions were performed in a total volume of 12 μl including 6 ng of DNA sample, primers at a final concentration of 300 nmol l^−1^ and 6 μl of SensiFAST™ SYBR^®^ Lo-ROX Kit (Bioline). We used Recombination Activating Gene 1 (*RAG1*) as a control SCG, verified using a BLAST analysis on the great tit genome. The gene *RAG1* was amplified using the primers RAG1 forward (5′-TCG GCT AAA CAG AGG TGT AAA G-3′) and RAG1 reverse (5′-CAG CTT GGT GCT GAG ATG TAT-3′). For mtDNA copy number, we used cytochrome oxidase subunit 2 (COI2) as a specific mitochondrial gene after verifying that it was not duplicated as a pseudo-gene in the nuclear genome, using a BLAST analysis on the great tit genome. We used the primer sequences COI2 forward (5′-CAAAGATATCGGCACCCTCTAC-3′) and COI2 reverse (3′-GCCTAGTTCTGCACGGATAAG-5′). Samples were run in triplicate. qPCR conditions were 3 min at 95°C (polymerase activation), followed by 40 cycles of 10 s at 95°C, 15 s at 58°C, 10 s at 72°C (DNA denaturation, primer annealing, DNA extension and fluorescence reading). The melting curve program was 15 s at 95°C, 1 min at 58°C, 0.1°C s^−1^ increase to 95°C, and then hold for 15 s at 95°C*.* A DNA sample prepared as a pool of DNA from 10 adult individuals was used as a reference sample (i.e. ratio=1.0 for mtDNA copy number) and was included in triplicate in every plate. qPCR efficiency (mean±s.d.) of control and mitochondrial genes was 91.4±0.003% and 104.5±0.005%, respectively. Repeatability of mtDNA copy number measurements estimated with sample triplicates was high (*R*=0.921, 95% confidence interval CI=[0.907; 0.934], *n*=1287). We also calculated the inter-plate repeatability of mtDNA copy number measurements using samples measured on different plates (*R*=0.867, 95% CI=[0.822, 0.916], *n*=211). All the qPCR assays (*n*=10 plates) were performed on a 384-QuantStudioTM 12 K Flex Real-Time PCR System (Thermo Fisher Scientific).

### Molecular sexing

Nestlings were molecularly sexed using a qPCR approach adapted from Chang et al. (2008) and Ellegren and Fridolfsson (1997), using blood samples when available (2 nestlings per brood). Forward and reverse sexing primers were 5′-CACTACAGGGAAAACTGTAC-3′ (2987F) and 5′-CCCCTTCAGGTTCTTTAAAA-3′ (3112R), respectively. qPCR reactions were performed in a total volume of 12 µl including 6 ng of DNA, primers at a final concentration of 800 nmol l^−1^ and 6 μl of SensiFAST™ SYBR^®^ Lo-ROX Kit (Bioline). qPCR conditions were: 3 min at 95°C, followed by 40 cycles of 45 s at 95°C, 60 s at 52°C and 60 s at 72°C, followed by a melting curve analysis (95°C 60 s, 45°C 50 s, increase to 95°C at 0.1°C s^−1^, 95°C 30 s). Samples were run in duplicate on a single plate and 6 adults of known sex were included as positive controls.

### Mitochondrial respiration

Mitochondrial respiration was analyzed using high-resolution respirometry (Oroboros Instruments, Innsbruck, Austria) at 40°C, adapted from the protocol described in Stier et al. (2019) (protocol modifications: mitochondrial respiration rate estimated using 30 µl of fresh blood when available, suspended in Mir05 buffer). We analyzed four mitochondrial respiration rates: (1) the endogenous cellular respiration rate before permeabilization (ROUTINE), (2) the maximum respiration rate fueled with exogenous substrates of complex I and II, as well as ADP (CI+II), (3) the respiration rate contributing to proton leak (LEAK, i.e. not producing ATP but dissipating heat), and (4) the respiration rate supporting ATP synthesis through oxidative phosphorylation (OXPHOS). We also calculated two mitochondrial flux ratios (flux control ratio, FCR): (1) OXPHOS coupling efficiency*=*(1−LEAK)/CI+II), and (2) the proportion of maximal respiration capacity used under endogenous cellular conditions (i.e. ROUTINE/CI+II FCR). The former provides an index of mitochondrial efficiency in producing ATP, whereas the latter reflects the cellular control of mitochondrial respiration by endogenous ADP/ATP turnover and substrate availability. Because of the logistical constraints of respirometry measurements (i.e. the need to work on freshly collected samples, >2 h of processing per 2 samples), the analysis of mitochondrial respiration was limited to one nestling per nest (repeated measurements from the same individuals at day 7 and day 14), summing up to 89 samples from 48 individuals (sample sizes at day 7 and day 14, respectively: *n*_control_=11/11, *n*_CORT_=11/10, *n*_TH_=14/12, *n*_CORT+TH_=10/10). Mitochondrial respiration rates were not analyzed from juveniles because of logistical constraints. The technical repeatability of mitochondrial respiration measurements was high: ROUTINE: *R*=0.989 (95% CI=[0.957, 0.997]); CI+CII: *R*=0.992 (95% CI=[0.968, 0.998]); LEAK: *R*=0.982 (95% CI=[0.929, 0.995]); OXPHOS: *R*=0.992 (95% CI=[0.968, 0.998]), based on *n*=9 duplicates.

### Statistical analyses

Statistical analyses were conducted using R v.4.0.2 (http://www.R-project.org/). To test for the effects of prenatal hormones on bird development, mitochondrial function and survival, we considered CORT and TH treatments (as separate 2-level factors: CORT yes/no and TH yes/no) and their interactions as fixed factors. In other words, as a 2-level treatment, the CORT group included all groups that received a CORT treatment, i.e. CORT and CORT+TH, and the non-CORT group included all groups that did not received a CORT treatment, i.e. TH and control. The same applied for TH groups, i.e. TH and CORT+TH, and non-TH groups, i.e. CORT and control. Non-significant terms were dropped (starting with interactions) in a backward-stepwise procedure to obtain the lowest Akaike information criterion (AIC) value. The effects of CORT and TH treatment on survival metrics (hatching success, fledging success and recapture probability in the autumn of 2019 and 2020) were evaluated using generalized linear mixed models (GLMM), with logistic binary distributions of the dependent variables (survival: 0=dead, 1=alive). Nest box ID was considered as a random intercept to account for the non-independence of nestlings reared in the same conditions, except for the recapture probability as adults because we did not recapture enough individuals per nest. We tested the effects of CORT and TH treatment on development time (incubation time per nest) using a linear model (LM).

The effect of CORT and TH treatment on growth metrics was analyzed in two steps. We first tested treatment effects on postnatal body mass growth (day 2, day 7, day 14) using a linear mixed model (LMM) with nest box ID and bird ID as random intercepts, to account for repeated measures on individual offspring and non-independence of nestlings reared in the same conditions. To test for differences in body mass gain, we also tested the effects of CORT and TH treatment at each age (day 7, day 14 and in juveniles – autumn 2019) on body mass, while controlling for the previous body mass as a covariate in separate LMMs with nest box ID specified as a random intercept. We analyzed body size (using wing length as a response variable) and body condition (i.e. body mass controlled for the wing length) at each age using LMMs with nest box ID specified as a random intercept.

mtDNA copy number data distribution did not fulfill the criteria of normality according to a Cullen and Frey plot (‘fitdistrplus’ package; Delignette-Muller and Dutang, 2015); therefore, we evaluated the effects of CORT and TH treatment on mtDNA copy number using a GLMM (gamma error distribution, log link). We included nest box ID as a random intercept and bird ID as a repeated factor to account for the non-independence of measures from the same individual. All mitochondria respiration rates (recorded at day 7 and day 14; including ROUTINE, LEAK, OXPHOS, CI+II) were tested with LMMs. We analyzed mitochondrial respiration rate at both the cellular level (i.e. respiration measurements expressed relative to cell number), which indicates respiration properties per unit of cells, and the mitochondrial level (i.e. respiration measurements controlled for mitochondrial density by inclusion of mtDNA copy number as a covariate), which indicates the respiration rate per unit of mitochondria. For models including repeated measures across time (body mass, mtDNA copy number, mitochondrial respiration measurements), we initially included CORT, TH, age and all interactions as fixed factors and removed non-significant interactions following a backward-stepwise procedure to obtain the lowest AIC value.

We also preliminarily included nestling sex as a fixed factor in our models to investigate sex-specific effects on growth metrics and mtDNA copy number. However, nestling sex never had a significant effect on morphometric traits and we decided to remove sex from the associated models to increase sample size (only 2 nestlings per nest were molecularly sexed through qPCR, while for growth we collected morphometrics measurements for the whole brood). For juveniles, all individuals were morphologically sexed and thus we also included sex, as well as its interaction with CORT and TH treatment.

In all models, hatching date and brood size at day 2 (both proxies of environmental conditions) were included as covariates (not scaled, except in the mtDNA copy number model  because of the convergence issue) when applicable as they are known to correlate with development, physiology and survival. Normality and homoscedasticity of the residuals were visually inspected (*Q–Q* plots). All models were performed using the ‘lme4’ package (Bates et al., 2015). Results from type III ANOVA tables with *F*-values (or χ^2^ for GLMM) and *P*-values (i.e. testing the main effect of each factor and interaction) calculated based on Satterthwaite's method are presented in the text, and model estimates (with associated 95% CI and *P*-values) are reported in the tables. The package ‘emmeans’ was used for conducting multiple *post hoc* comparisons [adjusted with Tukey honest significant differences (HSD) correction] and estimating least-square means (lsmeans) ±s.e. as well as standardized effect sizes (https://github.com/rvlenth/emmeans). Results are given as means±s.e.m. Values were considered as statistically significant for *P*<0.05*.*

### Ethics

All procedures were approved by the Animal Experiment Committee of the State Provincial Office of Southern Finland (license no. ESAVI/5718/2019) and by the Environmental Center of Southwestern Finland (license no. VARELY/924/2019) granted to S.R.

## RESULTS

### Prenatal hormone effects on hatching, fledging success and development time

Hatching success (control 55.6%, CORT 53.4%, TH 62.7%, CORT+TH 58.6%) and fledging success (control 90%, CORT 89.8%, TH 75.7%, CORT+TH 74.4%) were not significantly affected by the prenatal hormone manipulation (GLMMs, all χ^2^<2.5, all *P*>0.11). Development time was significantly increased (+7%) by prenatal CORT supplementation (LM, CORT versus non-CORT: lsmean±s.e.: 12.8±0.2 versus 12.0±0.2 days, *F*_1,49_=6.27, *P*=0.015), but significantly decreased (−5%) by prenatal TH supplementation (TH versus non-TH: lsmean±s.e.: 12.1±0.2 versus 12.7±0.2 days; *F*_1,49_=4.26, *P=*0.044). However, there was no significant CORT×TH interaction (*F*_1,49_=2.24, *P*=0.14).

### Prenatal hormone effects on mitochondrial density

**Fig. 3.**
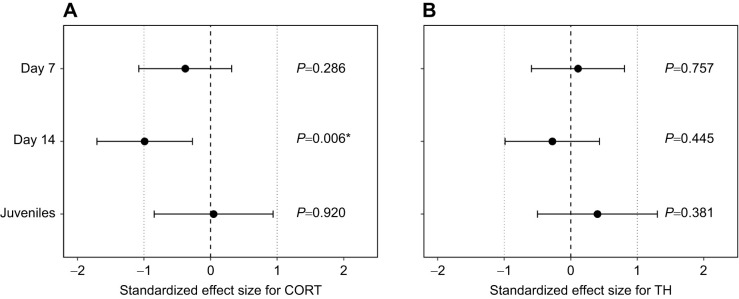
**Effects of prenatal CORT and TH treatment on mitochondrial density.** Mitochondrial density was measured at day 7 (*n*=99), day 14 (*n*=93) and juvenile age (*n*=37) (*n*=100 individuals in total) after (A) CORT and (B) TH treatment. Standardized effect sizes based on predicted values of the model are reported with their 95% confidence interval (CI). The age×CORT interaction was significant (χ^2^*=*8.65, *P*=0.013), and *post hoc* tests revealed a significant effect of CORT at day 14 only (*P=0.006).

We found a significant effect of prenatal CORT supplementation on the interaction between age and mitochondrial density (overall test for age×CORT: χ^2^*=*8.65, *P*=0.013; Fig. 3A). Mitochondrial density was significantly influenced by age (χ^2^*=*451.7, *P*<0.001), decreasing from day 7 to day 14 (Tukey HSD *post hoc*: *P*<0.001) and from day 14 to the juvenile stage (Tukey HSD *post hoc*: *P*<0.001; see Table 1 for estimates of final model). While prenatal CORT did not significantly affect mitochondrial density at day 7 (Tukey HSD *post hoc*: *P*=0.29) or in juveniles (Tukey HSD *post hoc*: *P*=0.92), it significantly decreased mitochondrial density by 27% at day 14 (Tukey HSD *post hoc*: *P*=0.006; Fig. 3A). We found no significant evidence for an effect of prenatal TH supplementation on mitochondrial density (χ^2^*=*0.003, *P*=0.96; Fig. 3B), nor for an interaction between prenatal TH and CORT (χ^2^*=*0.006, *P*=0.81). Brood size was negatively related to mitochondrial density (χ^2^*=*4.31, *P*=0.036), while hatching date was not significantly related to mitochondrial density (χ^2^*=*1.50, *P*=0.22; Table 1).

**Table 1.**
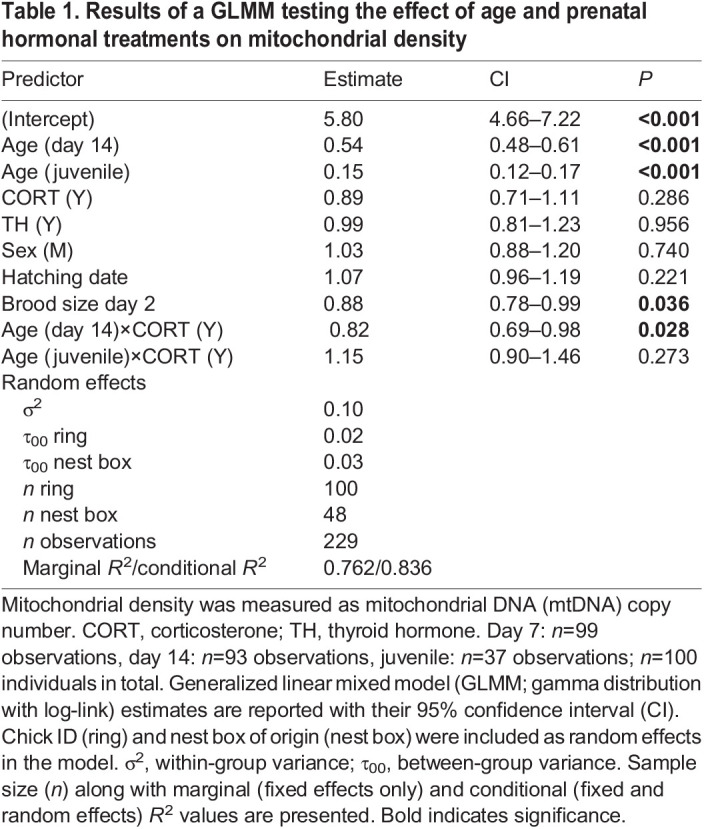
Results of a GLMM testing the effect of age and prenatal hormonal treatments on mitochondrial density

### Prenatal hormone effects on mitochondrial aerobic metabolism

**Fig. 4.**
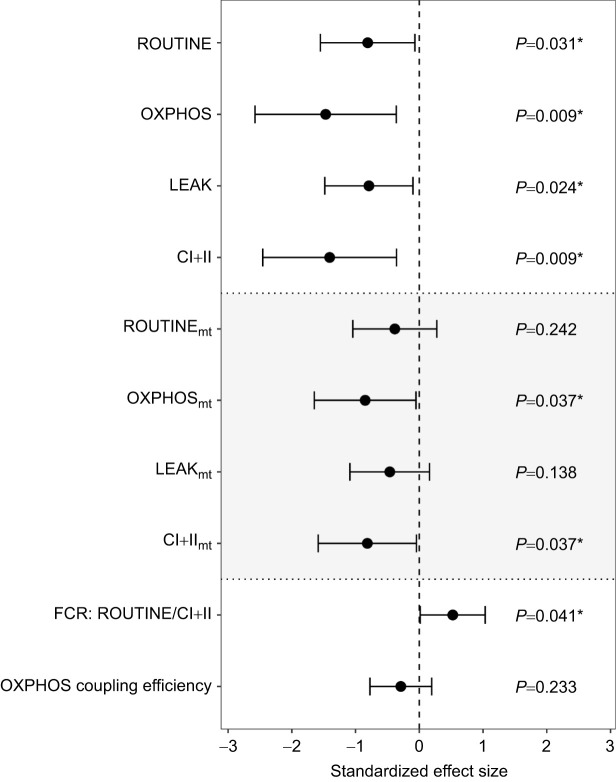
**Effect of a prenatal CORT treatment on mitochondrial aerobic metabolism.** Measurements were made on day 7 (*n*_CORT/non-CORT_=21/25) and day 14 (*n*_CORT/non-CORT_=20/23 individuals). Standardized effect sizes based on predicted values of the model are reported with their 95% CI. Response variables with subscript mt were corrected for mitochondrial density (mtDNA copy number included as a covariate in models). Age×CORT interactions were not statistically significant. Asterisks indicate significance.

Prenatal CORT supplementation significantly decreased all mitochondrial respiration rates measured at the cellular level (LMM: ROUTINE −15.8%, LEAK −16.4%, OXPHOS −22.9%, CI+II −21.7%; all *F*>4.2, all *P*<0.05; Fig. 4), in a similar way at both day 7 and day 14 (LMM, age×CORT interactions not statistically significant; all *F*<0.71, all *P*>0.41). Yet, all cellular respiration rates were positively associated with mitochondrial density (LMM; all *P<*0.001; Table 2). Controlling for mitochondrial density decreased the influence of prenatal CORT on respiration rate (i.e. respiration at the mitochondrial level), as evidenced by smaller effect sizes when correcting for mitochondrial density (Fig. 4; ROUTINE −6.5%, *F*=1.41, *P*=0.24; LEAK −9.8%, *F*=2.29, *P*=0.14; OXPHOS −14.2%, *F*=4.77, d.f.=30.65, *P*=0.037; CI+II −13.3%, *F*=4.72, d.f.=32.06, *P*=0.037; Table 2). Interestingly, nestlings from CORT-supplemented eggs had a significantly higher (+7.9%) usage of their mitochondrial maximal capacity (higher ROUTINE/CI+II FCR, *F*=4.79, d.f.=40.63, *P*=0.034; Fig. 4, Table 3), but we found no significant effect of prenatal CORT on OXPHOS coupling efficiency (OXPHOS coupling efficiency, *F*=1.32, d.f.=39.72, *P*=0.26; Fig. 4, Table 3).

**Table 2.**
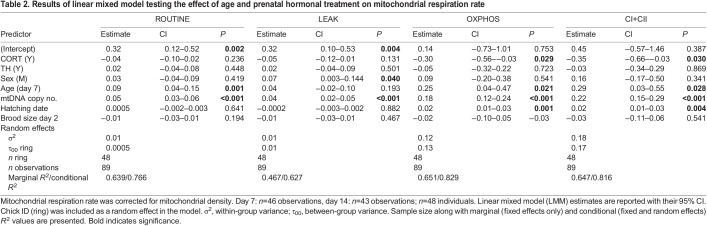
Results of linear mixed model testing the effect of age and prenatal hormonal treatment on mitochondrial respiration rate

**Table 3.**
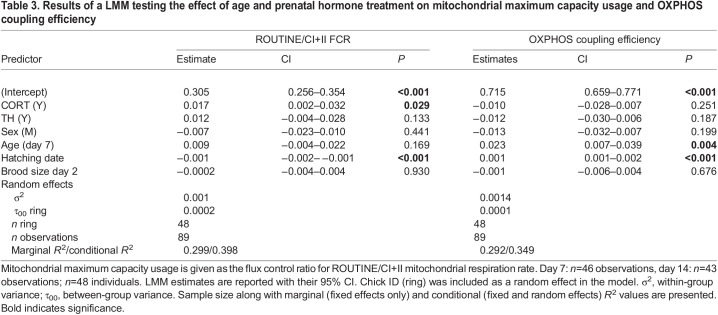
Results of a LMM testing the effect of age and prenatal hormone treatment on mitochondrial maximum capacity usage and OXPHOS coupling efficiency

Contrary to prenatal CORT treatment, there was no significant effect of prenatal TH supplementation on mitochondrial aerobic metabolism (LMM; all *F*<2.26, all *P>*0.14; Tables 2 and 3). All mitochondrial respiration rates significantly decreased between nestling day 7 and day 14 (LMM: ROUTINE −15.3%, OXPHOS −12.4%, CI+II −11.5%; all *F*>4.8, all *P*<0.032; Table 2), except LEAK (LMM; *F*=1.70, d.f.=67.93, *P*=0.20; Table 2). While ROUTINE/CI+II FCR was not significantly impacted by age (*F*=1.89, d.f.=44.42, *P*=0.18; Table 2), younger chicks had more efficient mitochondria (i.e. 2.9% higher OXPHOS coupling efficiency, *F*=8.33, d.f.=43.92, *P*=0.006; Table 3). Males showed a significantly higher LEAK (lsmean: +16.5%, *F*=4.23, d.f.=37.79, *P*=0.047) than females when controlling for mitochondrial density (Table 2), but we did not find other significant sex differences in mitochondrial aerobic metabolism (LMM; all *F*<1.65, all *P>*0.20; Table 2). Brood size was not significantly associated with mitochondrial aerobic metabolism traits (LMM; all *F*<1.69, all *P*>0.20; Tables 2 and 3). All mitochondrial aerobic metabolism traits except ROUTINE (*F*=0.22, d.f.=42.34, *P*=0.64) and LEAK (*F*=0.02, d.f.=40.89, *P*=0.88) were significantly positively associated with hatching date (LMM; all *F*>8.10, all *P*<0.008; Tables 2 and 3).

### Prenatal hormone effects on growth

**Fig. 5.**
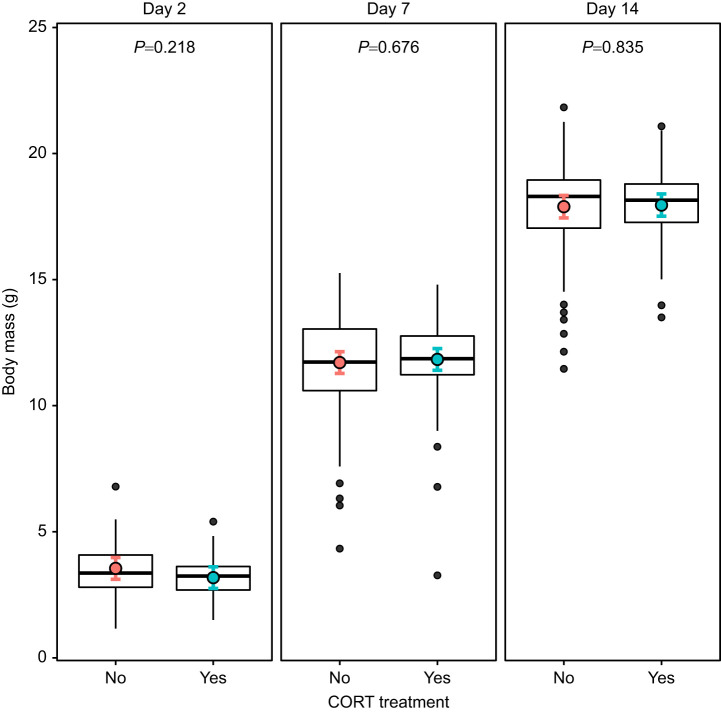
**Effects of prenatal CORT treatment on postnatal body mass.** Raw data for body mass distribution are plotted (day 2: *n*_CORT/non-CORT_=129/133; day 7: *n*_CORT/non-CORT_=123/128; day 14: *n*_CORT/non-CORT_=105/100 individuals) and least-square means (lsmeans) of the statistical model are presented as colored circles, with their 95% CI. The interaction age×CORT was statistically significant (overall test for the interaction *F*_2,460_=4.40, *P*=0.013), but none of the *post hoc* tests performed were significant (all *P*>0.18).

When analyzing body mass dynamics during postnatal growth (from day 2 to day 14), there was a significant interaction between age (day 2 versus day 7 versus day 14) and CORT treatment factors (*F*_2,460_=4.40, *P=*0.013; Table 4, Fig. 5), but no significant effect of prenatal TH supplementation (*F*_1,50_=0.95, *P=*0.33; Table 4)*.* Specifically, nestlings from CORT-supplemented eggs were slightly lighter (−11.3%) at day 2 than offspring from non-CORT-supplemented eggs (lsmean±s.e.: 3.54±0.22 versus 3.14±0.21 g), but reached the body mass of chicks from the non-CORT-supplemented group at day 7 and 14 (Fig. 5), although these differences were not statistically significant in *post hoc* analyses (Tukey HSD *post hoc*: all *P>*0.18).

**Table 4.**
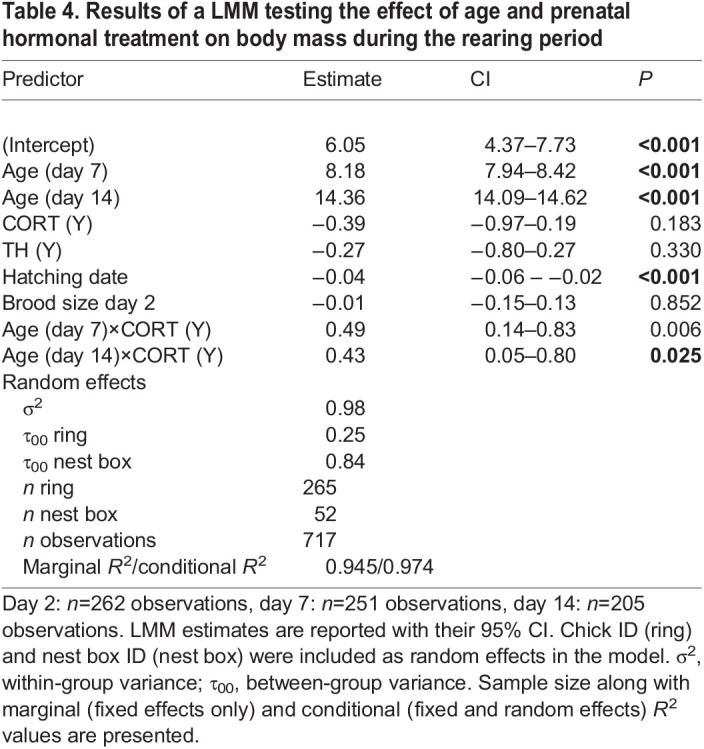
Results of a LMM testing the effect of age and prenatal hormonal treatment on body mass during the rearing period

Analyzing the different postnatal stages separately (day 2, day 7 and day 14) for body mass gain (i.e. body mass at time *t* analyzed with body mass at time *t*−1 as covariate), body size and body condition did not reveal any significant effect of prenatal hormone treatment (i.e. CORT and TH), either as main factors (all *F*<3.65, all *P*>0.06; Tables S1−S3) or in interaction (CORT×TH: all *F*<3.75, all *P*>0.05). Yet, there was a non-significant trend for CORT chicks to gain more body mass between day 2 and day 7 (*F*_1,43.7_=3.65, *P*=0.063; Table S2), and for an interaction between CORT and TH in explaining body size at day 7 (*F*_1,47_=3.74, *P*=0.059), with chicks that received both hormones having smaller wings than others (lsmeans±s.e.: CORT+TH: 18.5±0.7 mm; non-CORT/non-TH 19.9±0.7 mm; CORT/non-TH: 20.7±0.7 mm; TH/non-CORT: 20.4±0.7 mm).

**Fig. 6.**
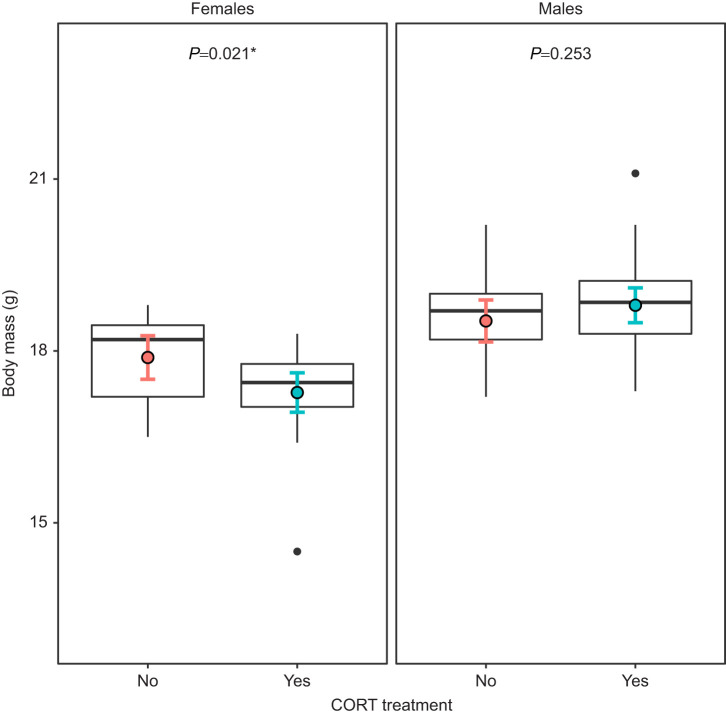
**Effects of prenatal CORT treatment and sex on juvenile body mass.** Raw data for body mass distribution are plotted (females: *n*_CORT/non-CORT_=26/19; males: *n*_CORT/non-CORT_=32/21 individuals) and lsmeans of the statistical model are presented as colored circles, with their 95% CI. The interaction CORT×sex was statistically significant (*F*=8.36, *P*=0.005). *P*-values of Tukey HSD *post hoc* tests are reported for each sex.

For juveniles (i.e. the subsample of individuals recaptured in autumn and morphologically sexed), we found a significant interaction between CORT treatment and sex on body mass (*F*=8.36, d.f.=40.89, *P*=0.005) and condition (*F*=8.91, d.f.=87.85, *P*=0.004) but not on body size (*F*=0.42, d.f.=82.66, *P*=0.52; Table S4). Body mass was 3.4% lower for females that received a prenatal CORT treatment than for females from the non-CORT group (*P=*0.021), while there was no significant effect of the prenatal CORT treatment on male body mass (*P=*0.25; Fig. 6). We found similar results for female body condition (CORT: −3.3%, *P*=0.016) and no significant differences between males (*P*=0.25). Prenatal TH supplementation did not significantly affect body mass, condition or size in juveniles (all *F*<0.33, all *P*>0.56; Table S4), nor in interaction with CORT treatment (CORT×TH: all *F*<4.06, all *P*>0.05).

### Prenatal hormone effect on recapture probability (i.e. proxy of apparent survival)

Recapture probability was not significantly affected by prenatal hormonal treatment either in the short term (juveniles in 2019: 56.03% and 42.34% for CORT versus non-CORT, χ^2^*=*2.35, *P*=0.12; and 50.00% and 48.62% for TH versus non-TH, χ^2^*=*0.01, *P*=0.93) or long term (adults in 2020: 15.52% and 10.81% for CORT versus non-CORT, χ^2^*=*0.68, *P*=0.41; and 15.25% and 11.01% for TH versus non-TH, χ^2^*=*0.59, *P*=0.44). There was no significant interaction between prenatal CORT and TH treatments on the aforementioned parameters (all χ^2^<0.56 and all *P>*0.45).

## DISCUSSION

We tested for potential developmental plasticity related to two prenatal hormones in a wild great tit population. By experimentally increasing yolk hormone content to simulate higher maternal deposition of these hormones in the eggs, we investigated the effects of GCs, THs and their interaction on offspring mitochondrial aerobic metabolism, development and survival. Development time was significantly increased by prenatal CORT supplementation, but significantly decreased by prenatal TH supplementation. Elevated prenatal CORT exposure significantly reduced mitochondrial density and respiration rates, without significantly affecting mitochondrial coupling efficiency (OXPHOS coupling efficiency). Interestingly, such downregulation of mitochondrial aerobic metabolism might have been partially compensated for by a higher usage of maximal mitochondrial capacity (i.e. higher ROUTINE/CI+II FCR). We did not find very clear effects of prenatal hormone treatment on growth patterns and recapture probability. Yet, nestlings hatched from CORT-injected eggs were lighter at day 2 and had a tendency to grow faster in early life (i.e. day 2 to day 7), although these differences were not statistically significant in our experiment, so effects of prenatal CORT on nestling body mass, size and condition should be considered with caution. Recaptured females from the CORT group were lighter and in worse condition than juvenile females from the non-CORT group, whereas we did not find a significant difference in males. Despite not being statistically significant, recapture probability was ca*.* 14% higher for juveniles from the CORT group. We expected prenatal TH treatment to promote mitochondrial biogenesis, leading to an increase in mitochondrial density and mitochondrial aerobic metabolism but found no support for this hypothesis. Other studies have also reported the lack of a significant effect of prenatal TH supplementation on nestling mitochondrial density in other avian species (Hsu et al., 2020, 2021 preprint; Stier et al., 2020). Several hypotheses may explain the contrasting results in studies focusing on maternal hormone effects, such as a specific dose-dependent or context-dependent response of maternal hormones, variation in initial hormones transferred/deposited by the mother or pleiotropic effects of maternal hormones (Groothuis et al., 2019). One limitation in the present study is the estimation of mitochondrial density and mitochondrial aerobic metabolism using blood cells. While it has been previously shown that mitochondrial function in blood cells is to some extent correlated to mitochondrial function in other tissues (Stier et al., 2017, 2022), TH may have tissue-specific effects that we were not able to detect in the present study.

Mitochondrial density was significantly reduced by a prenatal CORT increase, but in an age-specific manner as a significant effect was only observed at day 14 (a few days before fledging), suggesting that prenatal CORT had a delayed and transient effect (i.e. no evidence of developmental plasticity). This mitochondrial density reduction contributed to an apparent decrease of all respiration rates at the cellular level, including oxidative phosphorylation (as measured through OXPHOS). At the mitochondrial level (i.e. mitochondrial respiration rates corrected for mitochondrial density), CORT significantly decreased respiration related to both oxidative phosphorylation (OXPHOS) and maximal respiration capacity (CI+II). As the effect of prenatal CORT was consistent across time (i.e. at day 7 and 14, no significant age×CORT interaction), it is possible that prenatal CORT induced proper developmental plasticity, although effects later in life will have to be assessed to verify this hypothesis. Because of a decrease in the maximum capacity of mitochondria in the CORT group, mitochondria in that group were functioning, on average, significantly closer to their metabolic maximum (as measured through a significant increase in ROUTINE/CI+II FCR), yet without any clear change in coupling efficiency (no significant effect on OXPHOS coupling efficiency). Therefore, the downregulation of mitochondrial density and aerobic metabolism might have been partially compensated for by a higher endogenous usage of maximal mitochondrial capacity, but not by an increase in coupling efficiency. This effect of prenatal CORT on blood cell aerobic metabolism is in sharp contrast with results from a recent study on the same species that experimentally increased CORT levels after hatching (Casagrande et al., 2020): postnatal CORT supplementation led to an increase in respiration rate linked to proton leak and a concomitant decrease in coupling efficiency (Casagrande et al., 2020). This suggests that the same hormone can have contrasting effects on mitochondrial aerobic metabolism depending on the timing of exposure. As an alternative to a direct effect of prenatal CORT on mitochondrial density, it is possible that the effect we observed could be related to an effect of prenatal CORT on blood cell maturation. To the best of our knowledge, there is no information on blood cell maturation related to prenatal CORT increase in avian species. Yet, it is known that prenatal GCs contribute to the maturation of erythropoiesis in mammals (Tang et al., 2011). According to our results and other related studies (Hsu et al., 2021 preprint; Stier et al., 2020), mitochondrial density in avian blood cells decreases sharply during postnatal development. Thus, if the effect of CORT we observed (i.e. decreased mitochondrial density at day 14) was related to an effect of prenatal CORT on blood cell maturation, it would probably mean that an increase in prenatal CORT can accelerate the maturation of blood cells.

Despite reduced mitochondrial density and lower mitochondrial aerobic metabolism, CORT-supplemented nestlings reached, on average, a fledging body mass, body size and body condition similar to that of non-CORT individuals. The CORT treatment may have led to lower energy requirements, enabling individuals to reach similar mass/size despite lower mitochondrial density and aerobic metabolism. An alternative hypothesis could be that CORT nestlings obtained more food from their parents, which would be in line with the known effect of CORT on nestling begging rate (e.g. Rubolini et al., 2005). An interesting aspect of our results is that we found a medium-term sex-specific effect of CORT treatment on juveniles the following autumn (i.e. 9–20 weeks after fledging). Prenatal CORT supplementation significantly decreased body mass and condition of juvenile females, suggesting that the treatment may lead to some delayed deleterious effects. However, the mechanisms underlying the delayed effect of CORT on body mass and condition at the juvenile stage remain unclear. Sex-specific effects of prenatal GCs on adult metabolism have recently been documented in laboratory conditions on mammalian models (Kroon et al., 2020; Ruiz et al., 2020). Thus, it is possible that the sex-specific effect observed here on body mass could be related to metabolic alterations at the juvenile stage. Further studies are needed to test this hypothesis, for instance by measuring the effect of prenatal CORT on both whole-body and mitochondrial aerobic metabolism at the juvenile stage.

Contrary to our expectations and what has been found in a previous study on the same population (Hsu et al., 2021 preprint), the prenatal increase of TH in our study did not affect nestling growth patterns. Several hypotheses may explain these contrasting results. The impact of prenatal TH supplementation may depend on the original amount of TH deposited in eggs, which in itself varies between individuals and environmental conditions, such as ambient temperature or food availability (Ruuskanen and Hsu, 2018). Also, the effect may depend on postnatal environmental conditions, as maternal effects are context dependent (Groothuis et al., 2020). It is also possible that TH impacted traits that we did not measure in this study (e.g. specific target tissues, behavioral strategies). In addition, all traits were measured post-hatching and prenatal TH effects may be not visible anymore after hatching. These hypotheses may also explain why we were not able to detect significant interactions (e.g. permissive, synergistic or antagonistic effects) between CORT and TH treatments, although there was a non-significant trend towards a negative effect of the interaction between prenatal CORT and TH on body size at day 7.

One illustration of potential direct prenatal impact of CORT and TH is the result we obtained regarding development time (i.e. incubation duration). We found that a prenatal increase of CORT levels increased development time *in ovo*, while an increase in prenatal TH levels decreased development time. It has previously been shown that an augmentation of TH *in ovo* may accelerate hatching (Hsu et al., 2017). Measuring mitochondrial aerobic metabolism during embryo development will be necessary to understand whether such effects on embryo growth might be mediated by mitochondrial metabolism. However, as we monitored the nest only once a day to determine hatching date, overall incubation duration was estimated with a potential error of ±1 day, meaning that this result should be interpreted with caution, but warrants further investigation. Understanding how effects on development time may carry over and affect post-hatching phenotypes also requires further investigation.

One objective of this study was to investigate the effects of both prenatal TH and CORT on offspring short- and long-term survival. Prenatal hormone treatment did not significantly affect recapture probability (a proxy of apparent survival) in the following autumns (juveniles captured in 2019 and adults captured in 2020) even if we found a significant negative impact of CORT on the body mass and body condition of juvenile females. Yet, recapture probability seemed to be higher for juveniles from the CORT group, calling for further studies on the mechanisms by which prenatal hormones may induce differences in medium-term survival. It is worth noting that our results are based on a moderate sample size (*N*≈200 per age group for phenotypic data, and *N*≈45 per age group for high-resolution respirometry) and that further exploration with larger samples may be necessary to strengthen our conclusions.

### Conclusion

Our experimental approach mimicking an increase in maternal hormone deposition in eggs showed that an increase in CORT exposure *in ovo* decreases postnatal mitochondrial density and metabolism in blood cells, without markedly affecting mitochondrial coupling efficiency or nestling growth patterns. As mitochondrial function is expected to be central in the nexus between development, growth and metabolism, exploring how variation in mitochondrial function modulates offspring phenotype and fitness-related traits would help us to better understand the pathways through which maternal effects (including maternal hormones) operate. Exploring the impacts of prenatal maternal hormones on offspring mitochondrial function offers a novel perspective in explaining variation in offspring growth trajectories. As prenatal effects may have long term-consequences into adulthood (Groothuis et al., 2019; Groothuis et al., 2020), and as we indeed found decreased body mass and condition of CORT-treated juvenile females in our study, further investigations should focus on the long-term effects of prenatal hormones on mitochondrial aerobic metabolism later in life (in juvenile and adult birds).

## Supplementary Material

10.1242/jexbio.243414_sup1 Supplementary information
