# Correction: Effect of prenatal glucocorticoids and thyroid hormones on developmental plasticity of mitochondrial aerobic metabolism, growth and survival: an experimental test in wild great tits

**DOI:** 10.1242/jeb.249355

**Published:** 2024-08-14

**Authors:** Nina Cossin-Sevrin, Bin-Yan Hsu, Coline Marciau, Vincent A. Viblanc, Suvi Ruuskanen, Antoine Stier

There were several errors in *J. Exp. Biol.* (2022) **225**, jeb243414 (https://doi.org/10.1242/jeb.243414).

In the Materials and Methods, there was an error in the OXPHOS coupling efficiency equation, which should be (1–LEAK/CI+II) and not (1–LEAK)/CI+II).

The authors also report an error regarding the model selection for OXPHOS coupling efficiency. OXPHOS coupling efficiency was significantly impacted by the interaction between the age and the prenatal corticosterone (CORT) treatment group (age×CORT: *F*=5.92, *P*=0.019) (interaction not included in the final model in the original publication). Whereas differences between treatment groups in mitochondrial metabolism efficiency were not significant in 7-day-old nestlings (Tukey HSD *post hoc*: *P*=0.25), individuals hatched from CORT-injected eggs had a lower mitochondrial metabolism efficiency (–3.7%) at 14 days post hatching compared with the non-CORT group (Tukey HSD *post hoc*: *P*=0.016).

At all ages, both the maximal respiration capacity (CI+II) and the oxidative phosphorylation (OXPHOS) were lower in chicks hatched from CORT-injected eggs. The authors provide supplemental information regarding OXPHOS coupling efficiency, which represents an index of mitochondrial efficiency in producing ATP. The significant interaction presented in this Correction revealed that in addition to a general decrease (independent from the age) in both CI+II and OXPHOS in individuals from the CORT group, OXPHOS coupling efficiency was significantly lower in chicks from the CORT group at 14 days post-hatching. Concomitant with a decrease in mitochondrial density (as reported in Fig. 3), chicks hatched from the CORT-injected eggs also had a lower metabolism efficiency at 14 days post-hatching. Despite a lower mitochondrial metabolism efficiency at the end of the growth period, growth patterns (body mass and size measured at day 14) and fledging success of the chicks hatched from the CORT-injected eggs were not significantly different from the control group (as reported in Fig. 5). Whereas the authors found a potential long-lasting effect of the prenatal CORT treatment on female body mass recaptured as juveniles, it is worth noting that the decrease in mitochondrial metabolism efficiency at day 14 was not sex-specific (*F*=1.48*, P*=0.23). A complementary figure illustrating the corrected results is shown here.

As a result of this analysis, the authors also added the explanatory phrase ‘Except for OXPHOS coupling efficiency’ to the legend for Fig. 4.

**Figure:**
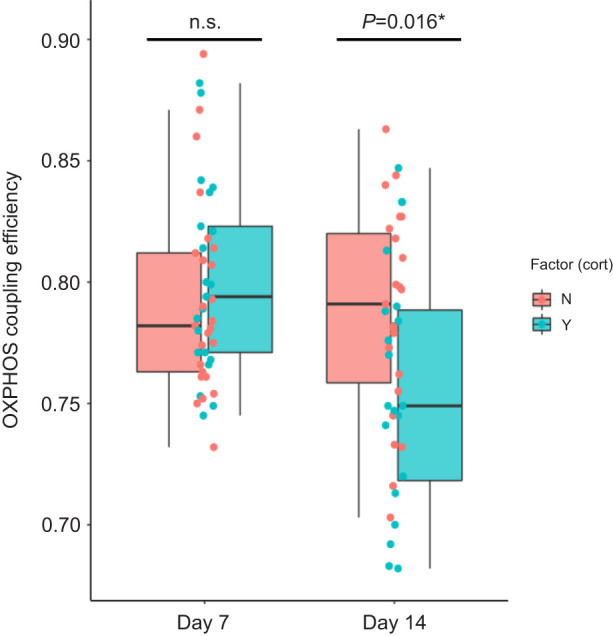
**Effect of prenatal CORT treatment on postnatal mitochondrial metabolism efficiency (OXPHOS coupling efficiency) according to the chick age.** Raw data for OXPHOS coupling efficiency are plotted (day 7: *n*_CORT/non-CORT_=21/25; day 14: *n*_CORT/non-CORT_=20/23 individuals). The interaction age×CORT was statistically significant (overall test for the interaction: *F*_1,42.68_=5.92, *P*=0.019). The hatching date, the brood size 2 days post hatching and the sex were included as covariates in the model, whereas the bird ID was included as random intercept. Statistics from the Tukey HSD *post hoc* comparisons are presented.

**Fig. 4 (new legend). Effect of a prenatal CORT treatment on mitochondrial aerobic metabolism.** Measurements were made on day 7 (*n*_CORT/non-CORT_=21/25) and day 14 (*n*_CORT/non-CORT_=20/23 individuals). Standardized effect sizes based on predicted values of the model are reported with their 95% CI. Response variables with subscript mt were corrected for mitochondrial density (mtDNA copy number included as a covariate in models). Except for OXPHOS coupling efficiency, age×CORT interactions were not statistically significant. Asterisks indicate significance.

The authors apologise for these errors, which do not have a major impact on the conclusions of the paper. The equation and the legend to Fig. 4 have been corrected in the online and PDF versions.

