# Supplementary material for: Effect of prenatal glucocorticoids and thyroid hormones on developmental plasticity of mitochondrial aerobic metabolism, growth and survival: an experimental test in wild great tits
Source: J Exp Biol. 2022 May 10;225(9):jeb243414. doi: 10.1242/jeb.243414 (PMC10216743; doi:10.1242/jeb.243414)
Supplement: Supplementary information [file jexbio-225-243414-s1.pdf]

Table S1. Results of linear mixed model testing the effect of prenatal hormonal treatments on body mass at day 2 post-hatching. Nest box identity (nestbox) was included as random effect in the model. σ2 = within-group variance; τ00 = between-group variance. Sample size along with marginal (fixed effects only) and conditional (fixed and random effects) R^2 are presented.

	Body mass day 2		
Predictors	*Estimates*	*CI*	*p*
(Intercept)	4.81	3.52 – 6.09	**<0.001**
CORT [Y]	-0.30	-0.73 – 0.12	0.165
TH [Y]	0.18	-0.25 – 0.62	0.403
brood size day 2	-0.02	-0.12 – 0.09	0.756
hatching date	-0.02	-0.04 – -0.01	**0.004**
**Random effects**			
σ2	0.27		
τ00 nestbox	0.53		
N nestbox	52		
Observations	262		
Marginal R2 / Conditional R2	0.119 / 0.705		

Table S2. Results of linear mixed models testing the effect of prenatal hormonal treatments on day 7: a. body mass gain (i.e. body mass at day 7 controlled for body mass at day 2); b. wing length (i.e. body size); and c. body condition (i.e. body mass corrected for body size). Nest box identity (nestbox) was included as random effect in the model. σ2 = within-group variance; τ00 = between-group variance. Sample size along with marginal (fixed effects only) and conditional (fixed and random effects) R^2 are presented.

**a.**	**Body mass day 7**		
Predictors	*Estimates*	*CI*	*p*
(Intercept)	6.32	4.38 – 8.27	**<0.001**
CORT [Y]	0.58	-0.02 – 1.18	0.056
TH [Y]	-0.27	-0.88 – 0.35	0.391
mass day 2	1.64	1.50 – 1.78	**<0.001**
brood size day 2	0.07	-0.08 – 0.22	0.373
hatching date	-0.01	-0.03 – 0.01	0.400
**Random effects**			
σ2	0.30		
τ00 nestbox	1.01		
N nestbox	49		
Observations	248		
Marginal R2 / Conditional R2	0.623 / 0.914		

**b.**	**Wing length day 7**		
Predictors	*Estimates*	*CI*	*p*
(Intercept)	24.08	19.75 – 28.42	**<0.001**
CORT [Y]	-0.60	-1.98 – 0.78	0.393
TH [Y]	-0.73	-2.10 – 0.65	0.300
brood size day 2	0.17	-0.20 – 0.53	0.377
hatching date	-0.08	-0.13 – -0.03	**0.004**
**Random effects**			
σ2	4.83		
τ00 nestbox	4.73		
N nestbox	49		
Observations	251		
Marginal R2 / Conditional R2	0.118 / 0.555		

**c.**	**Body mass day 7**		
Predictors	*Estimates*	*CI*	*p*
(Intercept)	3.84	2.28 – 5.40	**<0.001**
CORT [Y]	0.30	-0.10 – 0.70	0.140
TH [Y]	-0.04	-0.44 – 0.36	0.839
wing length day 7	0.44	0.40 – 0.48	**<0.001**
brood size day 2	-0.06	-0.17 – 0.04	0.233
hatching date	-0.01	-0.02 – 0.01	0.255
**Random effects**			
σ2	0.48		
τ00 nestbox	0.37		
N nestbox	49		
Observations	251		
Marginal R2 / Conditional R2	0.708 / 0.835		

Journal of Experimental Biology • Supplementary information

Table S3. Results of linear mixed models testing the effect of prenatal hormonal treatments on day 14: a. body mass gain (i.e. body mass at day 14 controlled for body mass at day 7); b. wing length (i.e. body size); and c. body condition (i.e. body mass corrected for body size). Nest box identity (nestbox) was included as random effect in the model. σ2 = within-group variance; τ00 = between-group variance. Sample size along with marginal (fixed effects only) and conditional (fixed and random effects) R^2 are presented.

**a.**	**Body mass day 14**		
Predictors	*Estimates*	*CI*	*p*
(Intercept)	15.08	12.26 – 17.90	**<0.001**
CORT [Y]	0.24	-0.54 – 1.02	0.552
TH [Y]	0.15	-0.56 – 0.87	0.674
mass day 7	0.52	0.42 – 0.61	**<0.001**
brood size day 2	-0.15	-0.36 – 0.07	0.177
hatching date	-0.05	-0.08 – -0.01	**0.004**
**Random effects**			
σ2	0.61		
τ00 nestbox	1.37		
N nestbox	41		
Observations	204		
Marginal R2 / Conditional R2	0.385 / 0.811		

**b.**	**Wing length day 14**		
Predictors	*Estimates*	*CI*	*p*
(Intercept)	58.18	52.30 – 64.06	**<0.001**
CORT [Y]	-0.17	-2.01 – 1.66	0.852
TH [Y]	-1.13	-2.87 – 0.61	0.204
brood size day 2	0.20	-0.30 – 0.70	0.430
hatching date	-0.14	-0.21 – -0.07	**<0.001**
**Random effects**			
σ2	5.99		
τ00 nestbox	6.99		
N nestbox	41		
Observations	204		
Marginal R2 / Conditional R2	0.224 / 0.642		

Journal of Experimental Biology • Supplementary information

**c.**	**Body mass 14**		
Predictors	*Estimates*	*CI*	*p*
(Intercept)	10.11	6.34 – 13.88	**<0.001**
CORT [Y]	0.32	-0.41 – 1.06	0.390
TH [Y]	0.06	-0.64 – 0.75	0.876
wing length day 14	0.21	0.16 – 0.26	**<0.001**
brood size day 2	-0.19	-0.39 – 0.01	0.063
hatching date	-0.03	-0.06 – -0.001	**0.042**
**Random effects**			
σ2	0.76		
τ00 nestbox	1.18		
N nestbox	41		
Observations	204		
Marginal R2 / Conditional R2	0.339 / 0.740		

Table S4. Results of linear mixed models testing the effect of prenatal hormonal treatments on juvenile: a. body mass; b. wing length (i.e. body size); and c. body condition (i.e. body mass corrected for body size). Nest box identity (nestbox) was included as random effect in the model. σ2 = within-group variance; τ00 = between-group variance. Sample size along with marginal (fixed effects only) and conditional (fixed and random effects) R^2 are presented.

**a.**	**Body mass juvenile**		
Predictors	*Estimates*	*CI*	*p*
(Intercept)	18.32	17.03 – 19.61	**<0.001**
sex [M]	0.64	0.17 – 1.10	**0.009**
CORT [Y]	-0.61	-1.11 – -0.12	**0.019**
TH [Y]	-0.11	-0.48 – 0.26	0.569
hatching date	-0.01	-0.03 – 0.02	0.548
sex [M] * CORT [Y]	0.89	0.29 – 1.49	**0.005**
**Random effects**			
σ2	0.49		
τ00 nestbox	0.11		
N nestbox	36		
Observations	98		
Marginal R2 / Conditional R2	0.398 / 0.509		

**b.**	**Wing length juvenile**		
Predictors	*Estimates*	*CI*	*p*
(Intercept)	76.59	74.15 – 79.02	**<0.001**
CORT [Y]	0.13	-0.63 – 0.90	0.731
TH [Y]	-0.05	-0.82 – 0.73	0.904
sex [M]	2.70	2.20 – 3.21	**<0.001**
hatching date	-0.01	-0.06 – 0.03	0.508
**Random effects**			
σ2	1.33		
τ00 nestbox	0.74		
N nestbox	36		
Observations	98		
Marginal R2 / Conditional R2	0.471 / 0.660		

Journal of Experimental Biology • Supplementary information

c.	Body mass juvenile		
Predictors	*Estimates*	*CI*	*p*
(Intercept)	12.40	3.60 – 21.20	**0.007**
sex [M]	0.41	-0.16 – 0.98	0.161
CORT [Y]	-0.64	-1.13 – -0.14	**0.015**
TH [Y]	-0.11	-0.48 – 0.27	0.573
wing length juvenile	0.08	-0.04 – 0.19	0.186
hatching date	-0.01	-0.03 – 0.02	0.620
sex [M] * CORT [Y]	0.91	0.31 – 1.51	**0.004**
**Random effects**			
σ2	0.48		
τ00 nestbox	0.11		
N nestbox	36		
Observations	98		
Marginal R2 / Conditional R2	0.407 / 0.520		

Journal of Experimental Biology • Supplementary information
